# A Systematic Review and Meta-Analysis of Epidemiology of Risky Sexual Behaviors in College and University Students in Ethiopia, 2018

**DOI:** 10.1155/2019/4852130

**Published:** 2019-03-20

**Authors:** Tadele Amare, Tebikew Yeneabat, Yohannes Amare

**Affiliations:** ^1^Department of Psychiatry, College of Medicine and Health Science, University of Gondar, Gondar, Ethiopia; ^2^Department of Midwifery, College of Health Sciences, Debre Markos University, Debre Markos, Ethiopia; ^3^Department of Internal Medicine, College of Medicine and Health Science, University of Gondar, Gondar, Ethiopia

## Abstract

**Background:**

Risk of sexual ill-health occurs with the onset of unsafe sexual activity, mostly among the adolescents, and continues as long as the risky activities are engaged in. Globally, and in Africa, adolescent AIDS-related mortality among adolescents has been increasing. Therefore, a systematic review and meta-analysis of epidemiology of risky sexual behaviors in college and university students in Ethiopia is mandatory.

**Methods:**

We conducted extensive search of articles as indicated in the guideline of reporting systematic review and meta-analysis (PRISMA). Databases such as PubMed, Global Health, Africa-wides, Google advance search, Scopus, and EMBASE were accessed for literature search. The pooled estimated effect of epidemiology of risky sexual behaviors and associated factors were analyzed by using the random effects model meta-analysis and 95% CI was also considered. PROSPERO registration number is CRD42018109277.

**Result:**

A total of 18 studies with 10,218 participants were encompassed in this meta-analysis. The estimated pooled prevalence of risky sexual behaviors among college and university students was 41.62%. Being male [OR: 2.35, with 95% (CI; 1.20, 4.59)], alcohol use [OR: 2.68, with 95% CI; (1.67, 4.33)] and watching pornography [OR: 4.74, with 95% CI; (3.21, 7.00)] were positively associated with risky sexual behaviors.

**Conclusion and recommendation:**

Risky sexual behavior among students was high. Educational institutions should give special attention for male sex, alcohol user, and students who watch pornography.

## 1. Introduction

Risky sexual behavior is defined as unprotected vaginal, oral, or anal intercourse [1]. Risk of sexual ill-health occurs with the onset of unsafe sexual activity, mostly among the adolescents, and continues as long as the risky activities are engaged in. In worldwide, 14,000 per day are newly infected by HIV, more than 95% were in developing countries due to risky sexual behavior [2].

Globally, and in Africa, adolescent AIDS-related mortality among adolescents has been increasing [3].

Factors increasing young people's vulnerability to infection include poverty, lack of power in sexual relationships, violence, traditional customs such as early marriage and harmful sexual practices, and gender disparities. One result is the transactional nature of sexual relationships, where women or girls exchange sex for money, school tuition, food, or housing [2, 4].

Prevalence of risky sexual behaviors in college and university students were 26% in Uganda [5], 63% in Nigeria [6], and 63.9% in Botswana [7].

Reasons for risky sexual behavior were pleasure, curiosity, peer influence, and financial benefit [8, 9]. Approximately 19 million new STI cases occur each year: about half in young persons aged 15 to 24. About 750,000 teenagers become pregnant each year [10]. Early age of sexual debut has been leading with multiple risk behaviors, containing depression, lack of condom use, and alcohol and drug use [11]. Consequence of risky sexual behaviors unintended pregnancy, sexually transmitted infections, mental illness, suicide, abortion, and academic withdrawal or dismissal [12, 13].

Factors that associated with risky sexual behavior were drinking alcohol [14, 15], being male [16], peer pressure [17, 18], and poverty [18].

Although college and university students are at a critical period for incidence of sexual risk behaviors, still little attention is given. Therefore, the estimated pooled prevalence and associated factors in risky sexual behavior are crucial.

## 2. Methods

**Figure 1:**
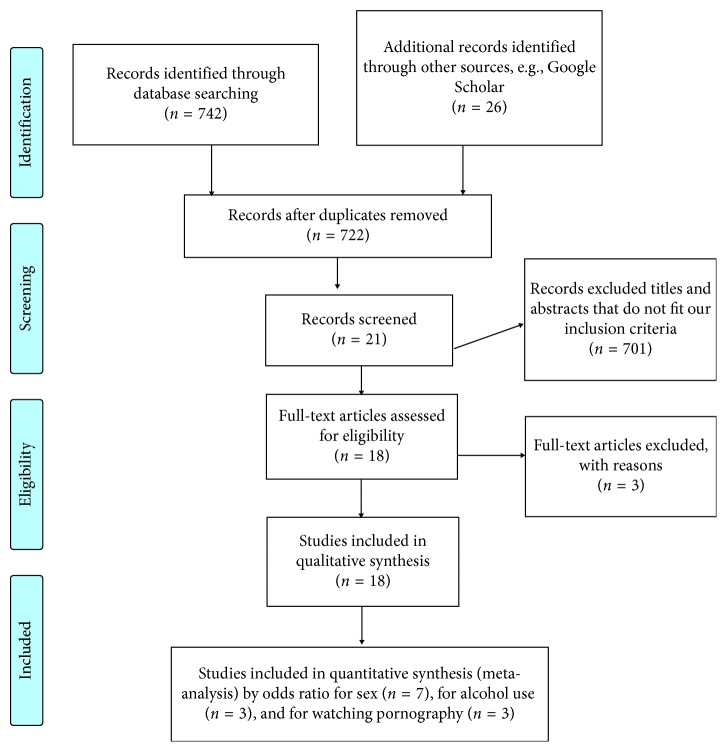
Flow chart showing how the research articles were searched, 2018.

We conducted extensive search of articles as indicated in the guideline of reporting systematic review and meta-analysis (PRISMA) [19]. Databases such as PubMed, Global Health, Africa-wides, Google advance search, Scopus, and EMBASE were accessed for literature search. We conducted our search in PubMed by using the following terms and keywords: “prevalence OR epidemiology OR magnitude OR incidence AND risky sexual behavior OR risky behavior AND associated factors OR predictors OR determinants OR risk factors AND college OR higher institution OR university AND students OR student OR learner OR learners AND Ethiopia OR Ethiopian.” For the other databases, we employed specific subjects heading as advising for each databases. Furthermore, to identify other related literatures, we manually searched the reference lists of eligible articles (Figure 1).

### 2.1. Eligible Criteria

Two reviewers (TA and TY) evaluated the relevant articles using their title and abstracts prior to retrieval of full-text articles. The retrieved full-text articles were further screened according to prespecified inclusion and exclusion criteria. To avoid selection bias, the Joanna Briggs Institute checklist for systematic reviews and research syntheses was used, which was scored nine out of eleven [20]. We resolved disagreements by discussing with a third reviewer (YA).

#### 2.1.1. Inclusion Criteria

  Study design type-cross-sectional
  Study subject-students in college and university
  Article published in English language
  Studies which reported magnitude of risky sexual behavior in college and university students
  Studies done in Ethiopia
  Study year from January, 2009 to August, 2018

#### 2.1.2. Exclusion Criteria

Letters, reviews, and international studies and duplicate studies were excluded.

### 2.2. Methods for Data Extraction and Quality Assessment

We used standardized data extraction form to extract data from identified studies. The following information was extracted for each included study: the name of the first author, publication date, study design, associated factors, sample size, study settings, confounders adjusted for risk estimate (OR), and the 95% confidence interval. Data extraction from source documents was done independently by three investigators. Disagreements were resolved by consensus.

The quality of included studies was evaluated by using the Newcastle–Ottawa Scale (NOS) [21]. Sample representativeness and size, comparability between participants, ascertainment of risky sexual behavior, and statistical quality were the domains of NOS uses to assess the quality of each study. Actual agreement and agreement beyond chance (unweighted Kappa) were used to evaluate the agreement among the three reviewers. We consider the value 0 as poor agreement, 0.01–0.20 as slight agreement, 0.21–0.40 as a fair agreement, 0.41–0.60 as moderate agreement, 0.61–0.80 as substantial agreement, and 0.81–1.00 as almost perfect agreement [22]. In this review, the actual agreement and agreement beyond chance was 0.82 which is almost perfect agreement.

### 2.3. Data Synthesis and Analysis

STATA version14 software was used for meta-analysis and forest plots that showed combined estimates with 95% CI. The overall pooled prevalence was estimated by random effect meta-analysis [23]. Heterogeneity was evaluated using *Q* statistic and the *I*^2^ statistics [23]. The magnitude of statistical heterogeneity between studies was assessed using *I*^2^ statistics and value of 25%, 50%, and 75% were considered to represent low, medium, and high respectively [24]. In this review data, the *I*^2^ statistics value was 97.1 with *p* value ≤ 0.001 which showed there was high heterogeneity. Therefore, a random effect model was used during analysis. Meta-regression was made to explore the probable source of heterogeneity. We also carried out a leave-one out sensitivity analysis to assess the key studies that exert major impact on between-study heterogeneity. Publication bias was assessed by funnel plot and Egger's regression test. There was no publication bias.

*Features of the studies*: all studies were comprised in Ethiopia. The study design of all research was cross-sectional and eighteen articles were included (Table 1).

## 3. Result

**Figure 2:**
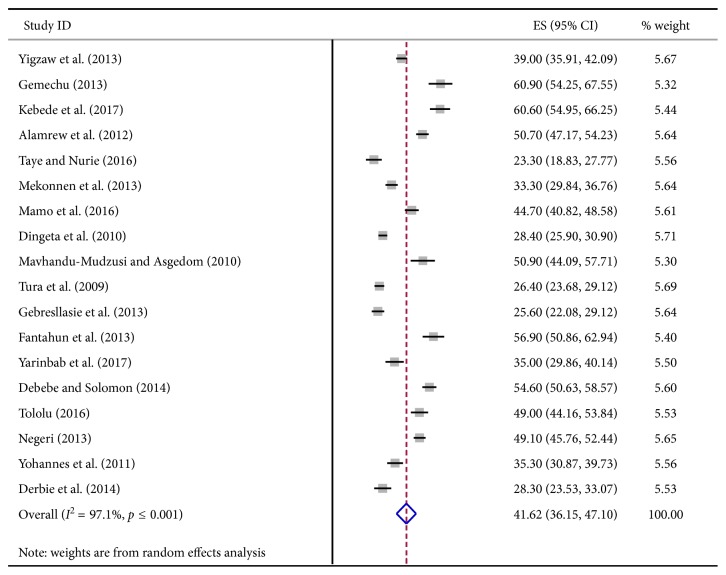
The pooled estimated prevalence of risky sexual behavior among college and university students, in Ethiopia 2018.

A total of 18 studies with 10,218 participants were comprised in this meta-analysis. According to different literatures in Ethiopia, the prevalence of risky sexual behavior ranged from 23.3% to 60.9%. The estimated pooled prevalence of risky sexual behaviors among college and university students was 41.62% with 95% CI (36.15, 47.10) (Figure 2).

### 3.1. Subgroup Analysis of the Prevalence of Risky Sexual Behavior in Students

**Figure 3:**
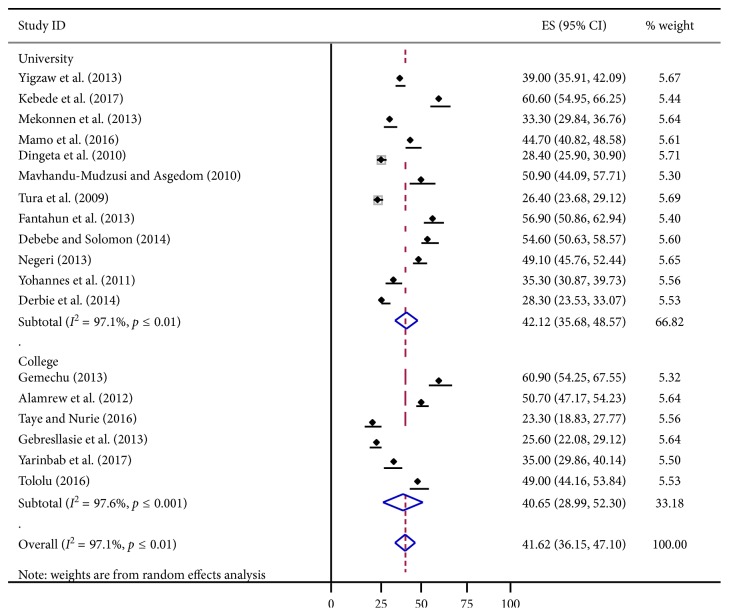
Forest plot presenting of subgroup analysis of pooled estimated prevalence of sexual behavior in college and university students, in Ethiopia, 2018.

From Figure 3 subgroup analysis were performed by the institution as possible source of heterogeneity between college and university. The estimated pooled prevalence of risky sexual behavior in college and university students were 40.65% and 42.12%, respectively.

### 3.2. Gender Difference and Risky Sexual Behaviors

**Figure 4:**
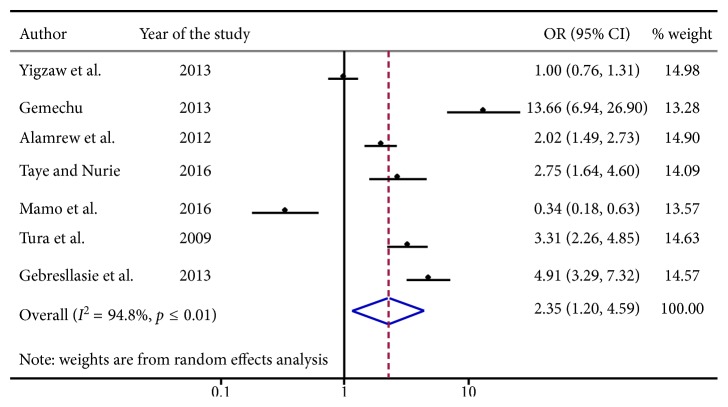
Forest plot presenting pooled random effect size (OR) of males related to females in risky sexual behavior among college and university students in Ethiopia, 2018.

From Figure 4 a total of seven articles were comprised in this analysis. There was a significant association between gender and risky sexual behaviors. Being male was 2.35 [OR: 2.35, with 95% (CI; 1.20, 4.59)] times more likely to engage in risky sexual practice compared to females.

### 3.3. Alcohol Use and Risky Sexual Behavior

**Figure 5:**
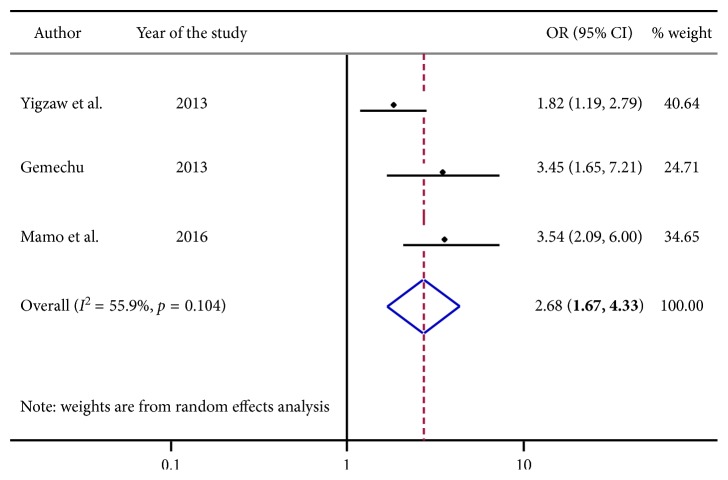
Forest plot presenting pooled random effect size (OR) of alcohol use related to nonalcohol use in risky sexual behavior among college and university students in Ethiopia, 2018.

From Figure 5, three articles were built-in in this analysis. Individuals who reported to have been influenced by alcohol for their risky sexual behavior practice were 2.68 [OR: 2.68, with 95% CI; (1.67, 4.33)] times more likely to engage in risky sexual practice.

### 3.4. Watching Pornography and Risky Sexual Behavior

**Figure 6:**
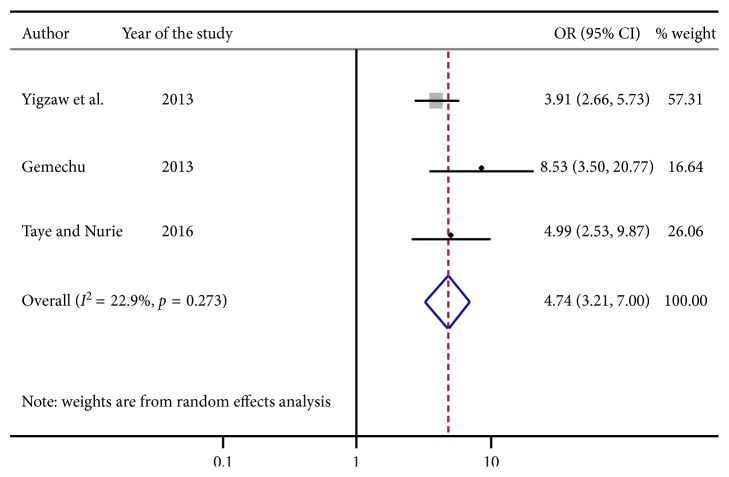
Forest plot presenting pooled random effect size (OR) of watching pornography related to nonwatching of pornography in risky sexual behavior among college and university students in Ethiopia, 2018.

From Figure 6 three articles were identified. Individuals who were watching pornography were about 5 [OR: 4.74, with 95% CI; (3.21, 7.00)] times more likely to engage in risky sexual practice than the counter parts.

## 4. Discussion

In this study, eighteen articles were included. Of these twelve studies were in university students whereas six were in college students. In Ethiopia, the prevalence of risky sexual behavior among college and university students ranged from 23.3% to 60.9%. The estimated pooled prevalence of risky sexual behavior among college and university students in Ethiopia were 40.65% (28.99, 52.30) and 42.41% (35.68, 48.57), respectively. The overall estimated pooled prevalence of risky sexual behavior was 41.62% (36.45, 47.10). This finding was lower than the study done in Nigeria [6] and Botswana [7]. However, this finding was higher than the study done in Uganda [5]. The difference might be sample size (in Uganda, the sample size was 261 which was small).

Factors that associated with risky sexual behavior among Ethiopian college and university students were being male was 2.35 [OR: 2.35, with 95% (CI; 1.20, 4.59)] times more likely to engage in risky sexual practice compared to females which was supported by [16]. Individuals who reported to have been influenced by alcohol for their risky sexual behavior practice were 2.68 [OR: 2.68, with 95% CI; (1.67, 4.33)] times more likely to engage in risky sexual practice which was supported by [14, 15]. Watching pornography was also risk factors for risky sexual behaviors. This might be watching pornography increases the motivation of sexual desire.

## 5. Conclusion and Recommendation

Risky sexual behavior among students was high. Educational institutions should give special attention for male sex, alcohol user, and students who watch pornography.

## Figures and Tables

**Table 1:** The prevalence of risky sexual behavior among students related to the institution, Ethiopia, 2018.

First author	Study year	Institution	Study design	Risky sexual behavior	Sample size	Cases	Prevalence (%)
Yigzaw et al. [25]	2013	Addis Ababa University	Cross-sectional	Yes	955	372	39
Gemechu [26]	2013	Alkan College	Cross-sectional	Yes	207	126	60.9
Kebede et al. [27]	2017	Aksum University	Cross-sectional	Yes	287	174	60.6
Alamrew et al. [28]	2012	Bahirdar Private College	Cross-sectional	Yes	771	391	50.7
Taye and Nurie [29]	2016	Bahirdar Regular Private College	Cross-sectional	Yes	344	80	23.3
Mekonnen et al. [30]	2013	Debre Markos College	Cross-sectional	Yes	714	238	33.3
Mamo et al. [31]	2016	Debre Markos University	Cross-sectional	Yes	631	282	44.7
Dingeta et al. [32]	2010	Haramaya University	Cross-sectional	Yes	1249	355	28.4
Mavhandu-Mudzusi and Asgedom [33]	2010	Jigjiga University	Cross-sectional	Yes	207	122	50.9
Tura et al. [34]	2009	Jimma University	Cross-sectional	Yes	1010	267	26.4
Gebresllasie et al. [35]	2013	Mekele College	Cross-sectional	Yes	590	151	25.6
Fantahun et al. [36]	2013	Mekele University	Cross-sectional	Yes	258	147	56.9
Yarinbab et al. [37]	2017	Mizantape College	Cross-sectional	Yes	331	116	35
Debebe and Solomon [38]	2014	Mada Walabu University	Cross-sectional	Yes	604	330	54.6
Tololu [39]	2016	Oromia Tevt College	Cross-sectional	Yes	410	201	49
Negeri [40]	2013	Wolega university	Cross-sectional	Yes	860	422	49.1
Yohannes et al. [41]	2011	Wolayta Sodo University	Cross-sectional	Yes	447	158	35.3
Derbie et al. [42]	2014	Debre Tabor University	Cross-sectional	Yes	343	97	28.3
